# ZRANB1 promotes cell proliferation and lymph node metastasis through SF3B3-mediated alternative splicing of CHEK2 in urothelial bladder cancer

**DOI:** 10.1038/s41419-026-08964-y

**Published:** 2026-06-09

**Authors:** Dong Yan, Wenjie Zhu, Yiran Tao, Lifang Huang, Yunlong Zhang, Wenliang Zhu, Qingqing He, Dejuan Wang, Jianguang Qiu

**Affiliations:** 1 Department of Urology, The Sixth Affiliated Hospital, Sun Yat-sen University, Guangzhou, China; 2 Guangdong Provincial Key Laboratory of Malignant Tumor Epigenetics and Gene Regulation, Sun Yat-sen Memorial Hospital, Sun Yat-sen University, Guangzhou, China; 3 Department of Urology, The Sixth Affiliated Hospital Yuexi Hospital/Xinyi People’s Hospital, Sun Yat-sen University, MaoMing, China; 4 Department of Urology, Sun Yat-sen Memorial Hospital, Sun Yat-sen University, Guangzhou, China

**Keywords:** Bladder cancer, Transcriptional regulatory elements

## Abstract

Urothelial bladder cancer (UBC) poses a considerable threat to public health, and its clinical management is limited by high recurrence rates and a tendency for progression. While dysregulation of the ubiquitin‒proteasome system (UPS) is a hallmark of tumorigenesis, the specific landscape of deubiquitinating enzymes (DUBs) in UBC remains largely underexplored. Multiple transcriptomic datasets were used for a comprehensive screening of ubiquitination-related genes in UBC, and ZRANB1 was identified as a potential oncogenic DUB, whose expression was validated using immunohistochemistry. High ZRANB1 expression was correlated with advanced pathological T stage, lymph node metastasis, and poor overall survival. The oncogenic role of ZRANB1 was assessed by proliferation, migration, and invasion assays in vitro, as well as in subcutaneous xenograft and lymph node metastasis models in vivo. Using immunoprecipitation coupled with mass spectrometry, we revealed that ZRNAB1 acted as a DUB to prevent the UPS-dependent degradation of SF3B3. The ZRANB1-SF3B3 axis subsequently modulates the alternative splicing of the cell cycle checkpoint kinase CHEK2, specifically inhibiting the production of the exon 4-skipped isoform (CHEK2-e4-). We demonstrated that while full-length CHEK2 is permissive for growth, the CHEK2-e4- isoform exerts a potent tumour-suppressive effect. This study reveals a novel post-translational mechanism linking the UPS to the RNA splicing machinery in UBC. ZRANB1 promotes tumorigenesis by stabilizing SF3B3 to prevent the generation of the tumour-suppressive CHEK2-e4- isoform, suggesting that ZRANB1 is a promising prognostic biomarker and therapeutic target.

## Introduction

Urothelial bladder cancer (UBC) is the most common malignancy of the urinary system and poses a considerable and growing threat to public health worldwide [1]. Approximately 614,000 newly diagnosed cases and 220,000 related deaths were reported in 2022 and the age-standardized incidence (ASIR) are predicted to continue to rise over the next decade [2, 3]. The clinical management of UBC is particularly burdensome because of its high likelihood of recurrence and progression. Approximately 75% of patients present with non-muscle-invasive bladder cancer (NMIBC) at diagnosis, which, despite favourable initial survival rates, requires lifelong surveillance via cystoscopic, radiological, and interventional procedures [4–6]. Consequently, UBC is associated with one of the highest lifetime treatment costs per patient among all cancer types, and the economic burden is likely to increase because of population ageing [7]. Approximately 15% to 20% of NMIBC cases progress to muscle-invasive bladder cancer (MIBC), with a median survival of ~15 months [8]. Despite advances in surgical techniques and immunotherapy, the prognosis for patients with advanced or metastatic disease remains poor, underscoring the urgent need to elucidate the molecular mechanisms driving UBC progression and to identify novel therapeutic targets [9].

Ubiquitin, a small protein consisting of 76 amino acids, is highly evolutionarily conserved and is found in all eukaryotic organisms [10]. Polyubiquitin or monoubiquitin forms covalent attachments to substrate proteins through ATP-dependent enzymatic cascades, including E1(activating enzyme), E2 (conjugating enzyme) and E3 (ligase) [11]. Ubiquitination modification is dynamic and reversible through the action of deubiquitinating enzymes (DUBs), which function as erasers of the ubiquitin code [12]. Ubiquitination can lead to diverse functional outcomes, such as signal transduction, regulation of subcellular localisation, and most commonly, protein degradation [13, 14]. The ubiquitin‒proteasome system (UPS) is the primary mechanism for intracellular protein homoeostasis, regulating the degradation of more than 80% of cellular proteins, especially short-lived and soluble misfolded/unfolded proteins [15, 16]. The specificity of this system relies on the finely balanced activities of E3 ubiquitin ligases and DUBs [17, 18]. Dysregulation of the UPS is a hallmark of tumorigenesis, leading to the aberrant stabilisation of oncoproteins or the excessive degradation of tumour suppressors. Emerging evidence suggests that DUBs play critical roles in cancer cell proliferation, metastasis, and chemotherapy resistance, making them attractive targets for drug discovery [19]. With increasing emphasis on the UPS and advanced understandings of ubiquitin modification, new methodologies, such as small-molecule inhibitor, protein-targeting chimeric molecules (PROTACs) and hydrophobicity tags (HyT), have been developed for tumour treatment [20]. However, the specific landscape of DUBs in UBC and their functional substrates remain largely underexplored.

In this study, we focused on ZRANB1 (Zinc Finger RANBP2-Type Containing 1), also known as TRABID, a DUB belonging to the OTU family. The human ZRANB1 protein comprises three N-terminal Npl4-like zinc finger (NZF) domains and one C-terminal OTU domain [21]. While ZRANB1 has been implicated in the regulation of ferroptotic resistance and stem–cell–like features in other malignancies, such as non–small–cell lung cancer and colorectal cancer, its function in UBC has not been previously characterised [22, 23]. Through a comprehensive screening of ubiquitination-related genes in multiple transcriptomic datasets, we identified ZRANB1 as a key factor in UBC tissues. Here, we report that ZRANB1 promotes UBC tumorigenesis by stabilising the splicing factor SF3B3 via deubiquitination. Furthermore, we demonstrate that the ZRANB1-SF3B3 axis modulates the alternative splicing (AS) of the cell cycle checkpoint kinase CHEK2, specifically by inhibiting the production of the tumour-suppressive CHEK2-e4- isoform. These findings reveal a novel post-translational mechanism linking the UPS to the RNA splicing machinery in UBC.

## Materials and methods

### Data screening

Four datasets and one predefined gene subset were used in the study, including GSE190079 (bladder cancer tumour tissues vs. adjacent non-tumour tissues control, *p* < 0.01, log_2_ | FC | > 0.3), GSE231383 (SV-HUC-1 vs. T24, UM-UC-3, J82 and 5637, *p* < 0.05, log_2_ | FC | > 0.5), GSE236932 (bladder carcinoma tissues vs. normal tissues, *p* < 0.01, log_2_ | FC | > 0.4), Gepia (TCGA + GTEx, tumour tissues vs. normal tissues, FDR < 0.05, log_2_ | FC | > 1) and a collection of ubiquitination-related genes (2 E1, 32 E2, 616 E3, and 91 DUB) as previously described [24].

### Patients and samples

To compare the ZRANB1 expression, tumour tissues and adjacent normal tissues from 12 UBC patients were obtained for Western blot analysis, and 15 tumour tissues with paired normal tissues were obtained for IHC staining. For survival analysis, a cohort of 110 UBC patients was included. All patients underwent surgery at Sun Yat-sen Memorial Hospital, Sun Yat-sen University, whose informed consent was obtained. The pathological diagnosis for all patients was confirmed by two independent pathologists, and the clinicopathological characteristics and ZRANB1 expression groups of the patients are summarised in Table 1.

**Table 1:** Correlation of ZRANB1 expression evaluated via IHC staining and UBC clinical parameters.

Characteristics		ZRANB1 expression		
	No. (%)	Low (%)	High (%)	*P*–value
Age				
<60	35	23	12	0.274
≥60	75	41	34	
Sex				
Male	97	57	40	0.736
Female	13	7	6	
T stage				0.009^**^
Ta-T1	47	34	13	
T2-T4	63	30	33	
LN status				0.008^**^
LN-	95	60	35	
LN+	15	4	11	
Grade				0.018^*^
Low	18	15	3	
High	92	49	43	
Total	110	64	46	

Chi-square test.

*P < 0.01.

**P < 0.05.

### Western blot analysis

Western blot analysis was conducted as previously described [25]. The primary antibodies used included anti-ZRANB1 (YT6929; Immunoway, USA), anti-GAPDH (AC001; ABclonal, China), anti-flag (#14793; CST, USA), anti-SF3B3 (YT4262; Immunoway, USA) and anti-ubiquitin (YM3636; ImmunoWay, USA). HRP-conjugated secondary antibodies (Goat Anti-Rabbit IgG, CWBIO, China) were applied as secondary antibodies. The full and uncropped western blots images were displayed in Supplementary files.

### Immunohistochemistry (IHC) and haematoxylin‒eosin (HE) staining

The tissue sections were formalin fixed, paraffin embedded and sectioned. For IHC staining, the sections were rehydrated and subjected to antigen retrieval with EDTA. Then the tissue sections were incubated with anti-ZRANB1 (PAB22260; Abnova, China), anti-SF3B3 (14577-1-AP; Proteintech, China) or anti-Ki-67 (27309-1-AP; Proteintech, China) antibodies at 4 °C overnight. The next day, the sections were incubated with horseradish peroxidase-conjugated secondary antibodies and stained with diaminobenzidine (DAB) and haematoxylin. The *H*-score was calculated based on the intensity of staining and the percentage of differently stained cells, as previously described [24]. For HE staining, the sections were dewaxed and dehydrated. Afterwards, the sections were treated with haematoxylin, differentiated with 1% acid alcohol, and blued with water. Following eosin staining, the sections were dehydrated and cleared.

### CCK-8, colony formation, transwell migration and invasion assays

CCK-8, colony formation, Transwell migration and invasion assays were conducted as previously described [26].

### Immunoprecipitation (IP) and mass spectrometry

Cells were lysed with cell lysis buffer for WB and IP (APE × BIO, USA) and then subjected to centrifugation. The supernatant was collected, 5% of which was taken as input. The magnetic beads were pre-coated with the indicated antibodies or IgG and incubated with the supernatant at 4 °C overnight. The next day, the beads were collected and washed, and the precipitated complexes were diluted and subjected to subsequent Western blot analysis or mass spectrometry. Mass spectrometry was performed at the Bioinformatics and Omics Centre, Sun Yat-Sen Memorial Hospital, Sun Yat-Sen University.

### Next-generation sequencing, RNA isolation, reverse transcription, real-time PCR (RT‒PCR) and nucleic acid electrophoresis

Next-generation sequencing was performed by NovelBio (China). Total RNA was extracted using an EZ-press RNA Purification Kit (EZBioscience, USA) according to the manufacturer’s protocol. Reverse transcription was conducted using HiScript IV All-in-One Ultra RT SuperMix for qPCR (R433-01; Vazyme, China). Briefly, 1000 ng of RNA and 5 μl of 4 × All-in-One Ultra qRT SuperMix were incubated at 50 °C for 5 min, followed by 85 °C for 5 s. RT‒PCR was performed with Hieff UNICON^®^ Power qPCR SYBR Green Master Mix (YEASEN, China) on a QuantStudio Dx instrument (Applied Biosystem, USA). The relevant abundance was calculated based on the cycle threshold (CT) value. Products from RT‒PCR were mixed with 6× loading buffer (TaKaRa, Japan) and GelRed (Biotium, US). Electrophoresis was conducted at a constant voltage in 1% agarose gel with TAE buffer, and the bands were visualised by UV irradiation. The primers used in this study were displayed in Supplementary Table 1.

### Animal experiments

The Animal Ethical and Welfare Committee of the Sixth Affiliated Hospital of Sun Yat-sen University approved all the procedures for the animal experiments in this study before the experiments were conducted. 4-week-old male BALB/c nude mice were purchased from GemPharmatech Co., Ltd and were randomly divided into NC and sh-ZRANB1 groups. Each group contained 5 mice in alliance with most studies. For the subcutaneous xenograft model, 5 × 10^6^ T24 cells were subcutaneously injected into the left flank of each mouse. Tumour size was measured every five days and tumour volume was calculated as tumour length × (tumour width)^2^/2. The mice were euthanized 30 days after the injection. The tumours were harvested, weighed and subjected to IHC and HE staining. For the LN metastasis model, 5×10^6^ T24 cells were suspended in 50 μl of PBS and injected into the right footpad of each mouse. The right popliteal LNs were collected 30 days after the injection and further subjected to weighing and HE staining.

### Statistical analysis

Statistical analysis was performed using SPSS 20.0 (IBM SPSS Statistics, USA). Data from three independent experiments are presented as mean ± standard deviation (SD) unless otherwise noted. Paired or unpaired Student’s *t*tests and Mann‒Whitney *U* tests were applied to compare the differences between two groups, depending on whether the data conformed to a normal distribution and whether the variance was similar between the groups. Chi-square tests and one-way analysis of variance (ANOVA) were used to assess the effects of multiple variables. For Kaplan–Meier survival analysis, the log-rank test was conducted. *P* < 0.05 was considered to indicate statistical significance.

## Results

### High expression of DUB ZRANB1 is related to poor survival in UBC patients

**Fig. 1:**
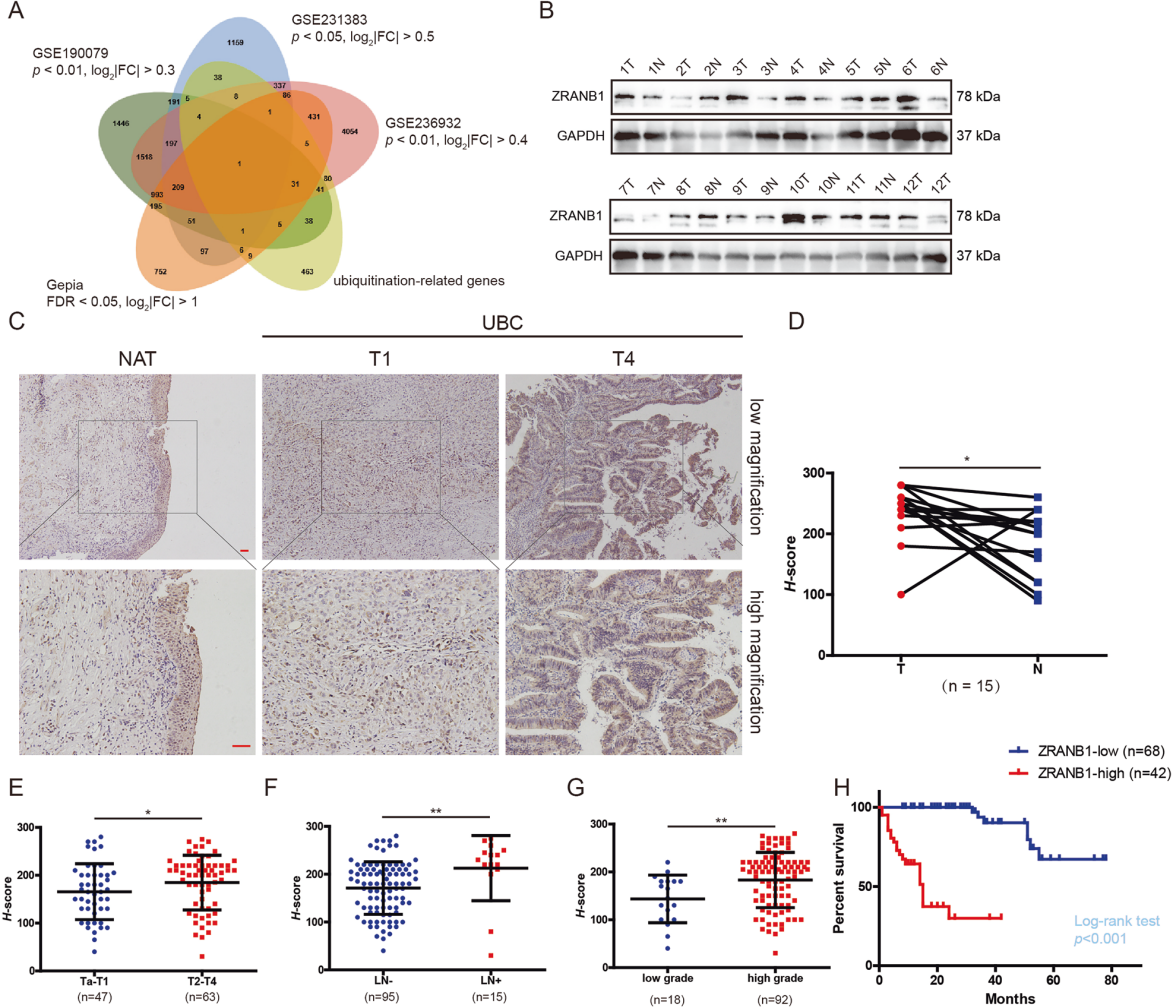
The overexpression of ZRANB1 is associated with poor survival of UBC patients. **A** The Venn diagram demonstrated a brief illustration of how ZRANB1 was screened out. **B** Western blot analysis of ZRANB1 expression in 12 UBC specimens (T) and matched normal adjacent tissues (N). **C** Representative images of UBC in different T stages and normal adjacent tissue (NAT) applied to IHC staining with anti-ZRANB1. **D** *H*-scores of 15 paired UBC specimens (T) and matched normal adjacent tissues (N), evaluated by IHC staining. Paired, two-tailed, Mann–Whitney *U* test. H-scores of ZRANB1 in 110 UBC specimens, compared by T stage (**E**), LN status (**F**), and pathological grade (**G**). Mann–Whitney *U* test. **H** 110 UBC specimens were divided into low or high ZRANB1 expression and the cutoff was determined by Youden’s Index. The Kaplan–Meier survival curve was plotted to compare the survival between two groups. Log-rank test. Data are shown as mean ± SD. *P < 0.05, **P < 0.01^.^ Scale bar = 100 μm.

To identify the key ubiquitination molecule in UBC, we defined a subset of 741 ubiquitination-related genes, including 2 E1, 32 E2, 616 E3, and 91 DUB, as previously described [24]. Afterwards, we cross-compared this subset with the Gepia and three GSE datasets (GSE190079, GSE231393, and GSE236932). Finally, ZRANB1 was the only gene identified for further investigation study (Fig. 1A).

To assess the clinical relevance of ZRANB1 expression in UBC patients, we detected the protein expression of ZRANB1 in 12 paired UBC tissues (T) and adjacent normal tissues (N) by Western blotting analysis, and found that ZRANB1 expression was increased in most UBC tissues (Fig. 1B). Moreover, we assessed the abundance of ZRANB1 in 15 UBC specimens along with their paired normal adjacent tissues and 110 UBC specimens with survival information via IHC analysis (Fig. 1C). It was found that ZRANB1 expression was higher in UBC tissues samples than in adjacent normal tissue samples (Fig. 1D), and higher ZRANB1 expression was associated with more advanced T stage, increased lymph node positivity, and a higher pathological stage (Fig. 1E‒G). Furthermore, higher abundance of ZRANB1 expression was associated with poorer overall survival in UBC patients (Fig. 1H).

### ZRANB1 influences the proliferation and invasion of UBC cells in vitro

**Fig. 2:**
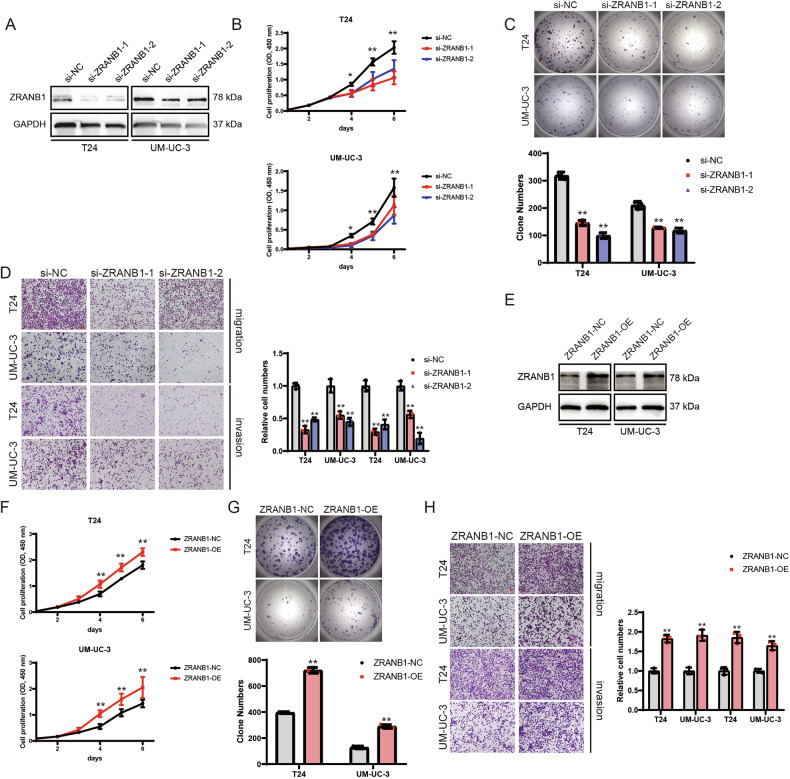
ZRANB1 promotes the proliferation and migration of UBC cells in vitro. **A** Western blot analysis showed the ZRANB1 expression in T24 and UM-UC-3 cell treated with siRNAs. **B** Cell viability of T24 and UM-UC-3 cells treated with ZRANB1 siRNAs, measured with CCK-8 assays. ANOVA. **C** Representative images and histogram analysis of colony formation assays with UBC cells silencing ZRANB1. Unpaired, two-tailed student’s *t*test. **D** Representative images and quantification of Transwell migration and invasion assays with T24 and UM-UC-3 cells treated with ZRANB1 siRNAs. Unpaired, two-tailed student’s *t*test. **E** The abundance of ZRANB1 in UBC cells infected with indicated lentivirus was measured via Western blot analysis. **F** Cell viability of T24 and UM-UC-3 cells overexpressing ZRANB1, measured with CCK-8 assays. ANOVA. **G** Representative images and histogram analysis of colony formation assays with UBC cells overexpressing ZRANB1. Unpaired, two-tailed student’s *t*test. **H** Transwell migration and invasion assays were performed in ZRANB1 overexpressing T24 and UM-UC-3 cells. Unpaired, two-tailed student’s *t*test. Data are shown as mean ± SD. *P < 0.05, **P < 0.01^.^ Scale bar = 100 μm.

To test the biological role of ZRANB1 in UBC, we transfected siRNAs into T24 and UM-UC-3 cells that specifically targeted ZRANB1. The silencing efficacy of the siRNAs was successfully validated by Western blotting analysis (Fig. 2A). Next, we evaluated the growth rate of UBC cells with ZRANB1 knockdown. CCK-8 assays revealed that silencing ZRANB1 expression significantly reduced the viability of T24 and UM-UC-3 cells (Fig. 2B). Cell colony formation assays demonstrated that ZRANB1 knockdown decreased the colony-forming ability of UBC cells (Fig. 2C). We also performed Transwell migration and invasion assays and found that ZRANB1 silencing inhibited the migration and invasion abilities of T24 and UM-UC-3 cells (Fig. 2D).

Afterwards, we cloned the ZRANB1 open reading frame (ORF) plus a 3x flag tag into the pLvx-puro plasmid, and ZRANB1-overexpressing cell lines were successfully constructed by lentivirus packaging, infection, and puromycin selection. The abundance of ZRANB1 was evaluated via Western blot analysis (Fig. 2E). Not surprisingly, elevated expression of ZRANB1 promoted the proliferation and colony-forming capacities of UBC cells (Fig. 2F‒G). Moreover, upregulated expression of ZRANB1 facilitated the migration and invasion of T24 and UM-UC-3 cells (Fig. 2H). Overall, our results demonstrate that ZRANB1 positively influences the growth and migration of UBC cells in vitro.

### ZRANB1 stabilizes SF3B3 through the ubiquitin–proteasome pathway

**Fig. 3:**
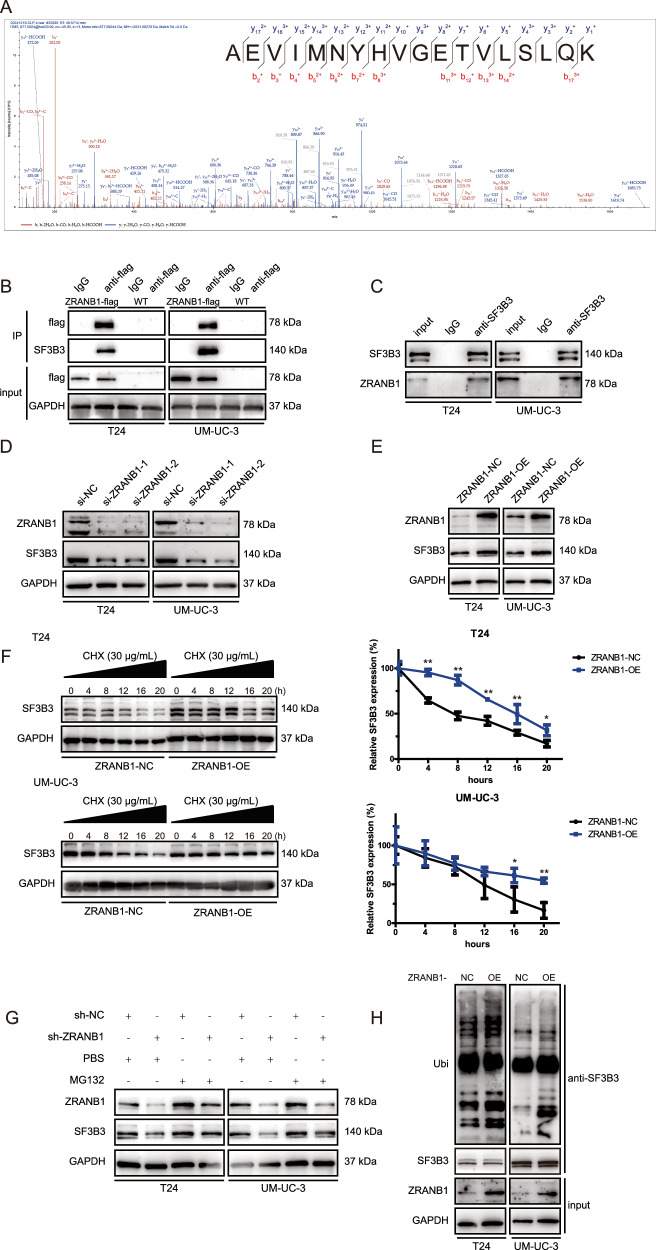
ZRANB1 interacts with SF3B3 to prevent ubiquitin mediated degradation. **A** The mass spectrum of SF3B3 identified in T24 cells exogenously expressing ZRANB1-flag fusion protein followed by co-IP with anti-flag. **B** UBC cells expressing ZRANB1-flag were applied to Western blot analysis after co-IP with anti-flag or IgG. **C** Co-IP in T24 and UM-UC-3 cells with indicated antibodies. The protein levels of ZRANB1 and SF3B3 were measured in UBC cells down-(**D**) or up-regulation (**E**) of ZRANB1, using Western blot analysis. **F** T24 and UM-UC-3 cells were subjected to CHX and then collected at the indicated time points, and relative SF3B3 expression was determined using Western blot analysis. The signal intensity was normalized to the intensity at the initial time. ANOVA. **G** Western blot analysis demonstrated the expression of ZRANB1 and SF3B3 in sh-NC or sh-ZRANB1 UBC cells treated with PBS or MG132. **H** Western blot analysis followed by co-IP evaluated the ubiquitination levels of UBC cells overexpressing ZRANB1. Data are shown as mean ± SD. *P < 0.05, **P < 0.01.

ZRANB1 was previously recognised as a deubiquitinase that plays diverse roles in multiple physiological and pathological processes [27, 28]. We intended to clarify its specific mechanism in UBC. First, we aimed to identify molecules that interact with ZRANB1. Because we failed to obtain satisfactory anti-ZRANB1 antibodies suitable for co-IP assays, we exogenously expressed ZRNAB1-flag fusion protein in T24 and UM-UC-3 cells. We subsequently conducted IP followed by mass spectrometry using anti-flag antibodies and IgG. A profile of the ZRANB1 interaction network was established, from which SF3B3 was selected for further investigation because of its high coverage and lack of relevant studies (Fig. 3A, Supplementary Table 2). To validate the binding between ZRANB1 and SF3B3, co-IP coupled with Western blotting analysis in UBC cells expressing ZRANB1-flag revealed that anti-flag antibody, but not IgG, enriched both ZRANB1 and SF3B3 (Fig. 3B). We also conducted co-IP coupled with Western blotting analysis in wild-type T24 and UM-UC-3 cells with an anti-SF3B3 antibody, which further validated our findings (Fig. 3C).

We subsequently investigated the regulatory relationship between ZRANB1 and SF3B3. When ZRANB1 expression was silenced, the abundance of SF3B3 protein was reduced while mRNA level was not significantly affected (Fig. 3D, Supplementary Fig. 1A). Conversely, over-expression of ZRANB1 in UBC cells led to an increase in SF3B3 protein level but did not affect its mRNA expression (Fig. 3E, Supplementary Fig. 1B). Since ZRANB1 generally functions as a deubiquitinase to influence target proteins posttranslationally, we applied CHX to halt translation in T24 and UM-UC-3 cells and measured the abundance of SF3B3 at the indicated time points. SF3B3 protein degradation occurred more slowly when ZRANB1 was overexpressed (Fig. 3F). We then investigated the effects of a series of inhibitors on this regulation, including SUMOylation inhibitor 2-D08, phosphatase inhibitor Calyculin A, histone methyltransferase inhibitor SGC707, palmitoylation inhibitor 2-BP, E1 Activating inhibitor PYR-41 as well as DMSO as negative control. It was observed that only PYR-41 blocked the effect of ZRANB1 overexpression on SF3B3 level (Supplementary Fig. 1C). To determine whether this regulation was dependent on the ubiquitin‒proteasome pathway, we treated cells with the proteasome inhibitor MG132. When MG132 was present, ZRANB1 silencing partly failed to reduce SF3B3 expression, suggesting that the proteasome pathway was involved (Fig. 3G). Finally, we measured the ubiquitination levels of SF3B3 in UBC cells overexpressing ZRANB1, and supplementation with ZRANB1 increased the ubiquitination of SF3B3 (Fig. 3H). Overall, these results demonstrated that ZRANB1 stabilizes SF3B3 through the ubiquitin–proteasome pathway.

### SF3B3 promotes carcinogenesis in UBC

**Fig. 4:**
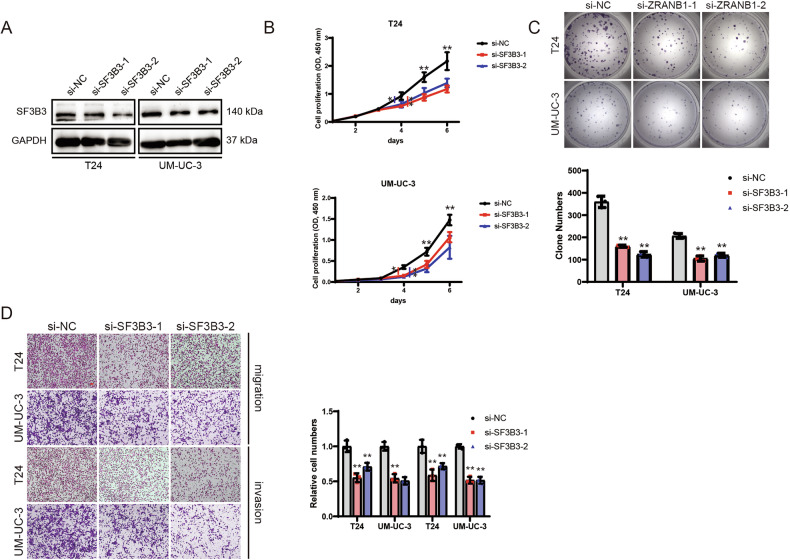
Silencing SF3B3 inhibits the proliferation and invasion of UBC cells in vitro. **A** The knockdown efficiency of siRNAs targeting SF3B3 in T24 and UM-UC-3 cells, validated by Western blot. **B** CCK-8 assays with UBC cells silencing SF3B3. ANOVA. **C** Representative images and quantification of colony formation assays with UBC cells downregulation of SF3B3. ANOVA. **D** Transwell migration and invasion assays evaluated the migration and invasion ability of UBC cells knocking down of SF3B3. ANOVA. Data are shown as mean ± SD. *P < 0.05, **P < 0.01^.^ Scale bar = 100 μm.

We subsequently aimed to understand the role of SF3B3 in UBC carcinogenesis. We transfected T24 and UM-UC-3 cells with siRNAs specifically targeting SF3B3, and the knockdown efficiency was verified by Western blot analysis (Fig. 4A). CCK-8 and colony formation assays demonstrated that reduced SF3B3 expression suppressed the viability and colony-forming ability of UBC cells (Fig. 4B, C). In accordance with ZRANB1 silencing, SF3B3 downregulation negatively influenced the migration and invasion of T24 and UM-UC-3 cells in vitro (Fig. 4D). Collectively, these findings suggest that SF3B3 plays an inhibitory role in UBC carcinogenesis.

### Overexpression of SF3B3 abrogates tumour-inhibition effects of ZRANB1 silencing in UBC cells

**Fig. 5:**
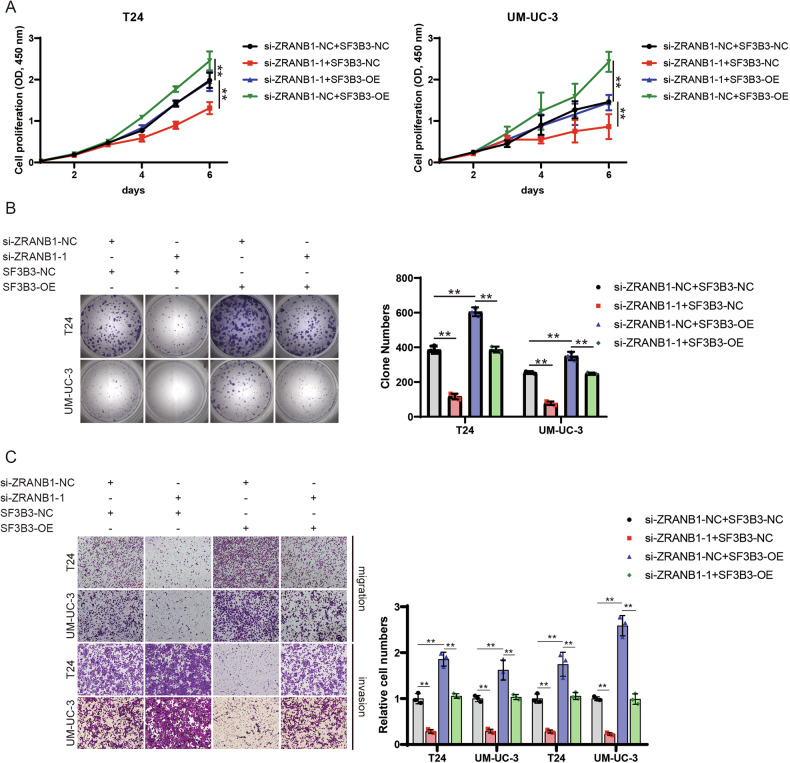
Supplementation of SF3B3 abolishes the antitumour effect brought by ZRANB1 silencing. **A**‒**C** UBC cells transfected with si-NC or si-ZRANB1-1 along with pcDNA3.1 plasmids containing an NC sequence or the SF3B3 ORF. The growth rate in vitro was evaluated by CCK-8 assays (**A**). The colony forming ability was measured by colony formation assays (**B**). The migration and invasion of T24 and UM-UC-3 cells were quantified by Transwell migration and invasion assays(**C**). ANOVA. Data are shown as mean ± SD. **P < 0.01. Scale bar = 100 μm.

Based on the data above, we hypothesised that ZRANB1 exerts its function through SF3B3. To test our hypothesis, we exogenously expressed SF3B3 in ZRANB1-NC and ZRANB1-silenced UBC cells. The results revealed that an increase in SF3B3 abundance in ZRANB1-NC cells increased proliferation, colony formation, migration and invasion abilities. However, when SF3B3 was supplemented in UBC cells in which ZRANB1 was knocked down, the inhibitory effects of ZRANB1 silencing on proliferation and invasion were abolished, suggesting that the antitumour effect of ZRANB1 inhibition was achieved through the downregulation of SF3B3 (Fig. 5A–C).

### SF3B3 is involved in the AS of CHEK2

**Fig. 6:**
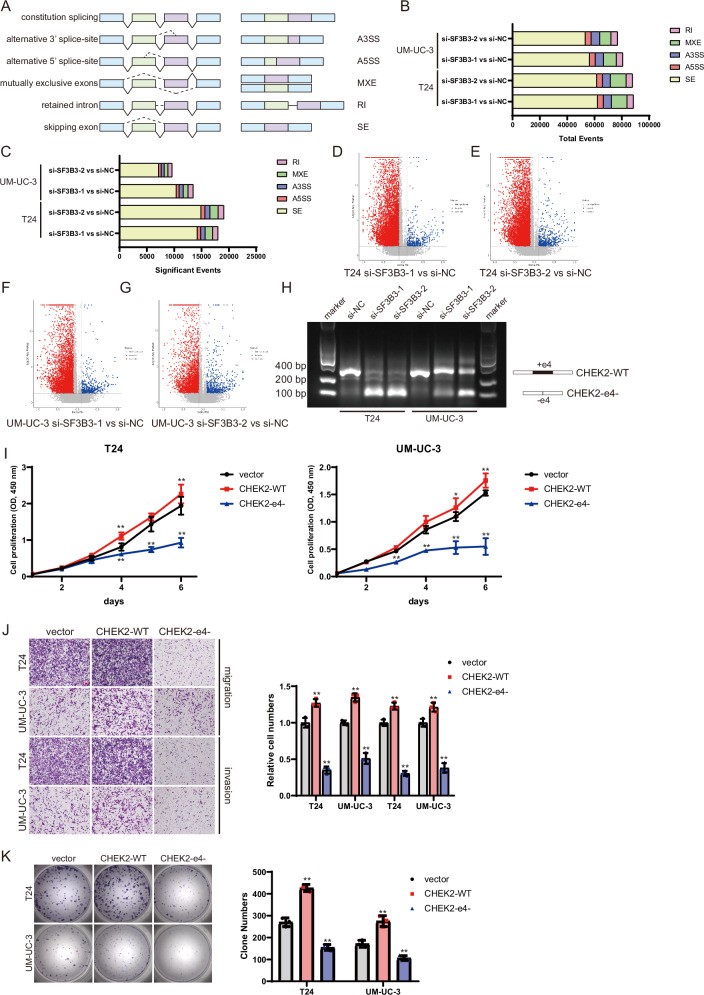
SF3B3 regulates the AS of CHEK2. **A** Illustrations of five most common categories of AS. Total (**B**) and significant (**C**) AS events identified via next-generation sequencing categorized into RI, MXE, A3SS, A5SS and SE. Volcano plots of SE events in T24 (**D**‒**E**) and UM-UC-3 (**F**‒**G**) cells silencing SF3B3. **H** Nucleic acid electrophoresis with specific primers targeting upstream and downstream of exon4 in *CHEK2*, performed with T24 and UM-UC-3 cells. **I** The growth rate of UBC cells expressing CHEK2-WT or CHEK2-e4- transcript, evaluated by CCK-8 assays. ANOVA. **J** The representative images and quantification of Transwell migration and invasion assays with T24 and UM-UC-3 cells supplementation with CHEK2-WT or CHEK2-e4-. ANOVA. **K** The representative images and quantification of colony formation assays with indicated cells. ANOVA. Data are shown as mean ± SD. *P < 0.05, **P < 0.01.

Many previous studies have reported that SF3B3 can regulate the AS of various transcripts, which naturally led us to investigate the impact of SF3B3 on AS in UBC [29, 30]. We performed next-generation sequencing on T24 and UM-UC-3 cells transfected with siRNAs targeting NC or SF3B3. The AS events were classified into five categories: alternative 3’ splice-site (A3SS), alternative 5’ splice-site (A5SS), mutually exclusive exons (MXE), retained intron (RI) and skipping exon (SE) (Fig. 6A). With respect to both total events and significant events, SEs were the most common events recognised (Fig. 6B, C). Moreover, more SE events were detected in NC cells than in SF3B3 knockdown cells, suggesting that SF3B3 primarily functions as an SE effector on most occasions (Fig. 6D–G). We selected the five most statistically significant SE events, including exon 28 skipping in DNAH14, exon 6 skipping in LIAS, exon 3 skipping in CCDC163, exon 2 skipping in PHF5A, and exon 4 skipping in CHEK2. We designed and synthesised pairs of primers targeting the upstream and downstream regions of skipping exons, and PCR was performed using cDNA from UBC cells with or without siRNA-mediated silencing of SF3B3. The PCR products were further subjected to nucleic acid electrophoresis, and after SF3B3 was knocked down, the shorter product (CHEK2-e4-) increased, whereas the longer product (CHEK2-WT) decreased, which indicated that SF3B3 acts as an essential inhibitor of exon 4 skipping in CHEK2 transcription (Fig. 6H, Supplementary Fig. 2).

CHEK2 is generally regarded as a tumour suppressor, and its variants are strongly associated with cancer risk [31]. Therefore, we intended to elucidate the functional roles of CHEK2-WT and CHEK2-e4- in UBC cells. Exogenous expression of CHEK2-WT slightly increased cell viability, colony formation, migration and invasion in both T24 and UM-UC-3 cells, while supplementation with CHEK2-e4- robustly suppressed the proliferation and motility of UBC cells (Fig. 6I–K). Collectively, these results suggest that SF3B3 is essential for the maintenance of full-length CHEK2 transcription, as it inhibited the AS of exon 4 to exert antitumour effect.

### ZRABN1 silencing inhibited the proliferation and lymph node metastasis of UBC cells in vivo

**Fig. 7:**
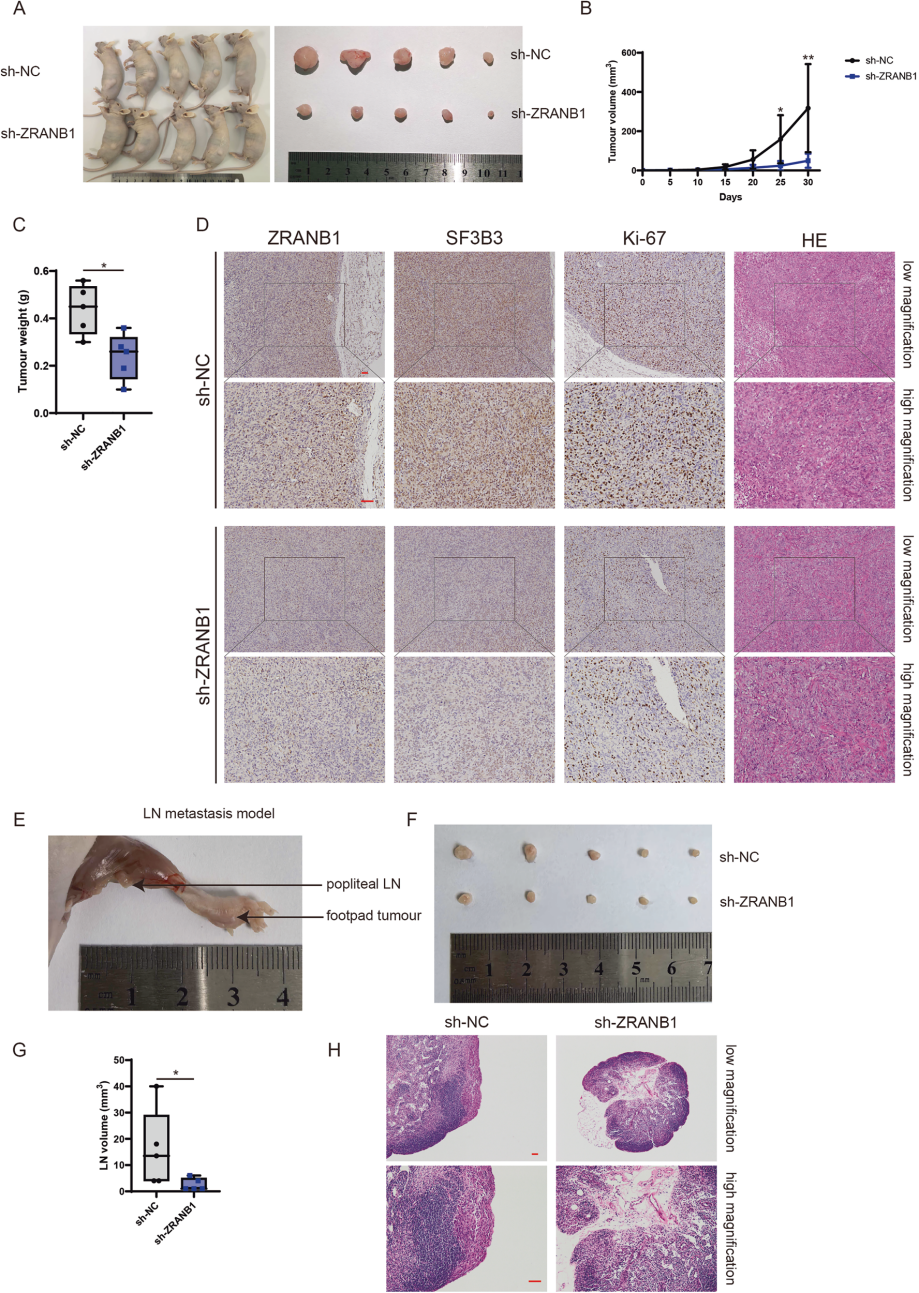
ZRANB1 promotes proliferation and LN metastasis of UBC cells in vivo. **A** The overview(left) and tumours(right) of subcutaneous xenograft model. 5×10^6^ T24 cells were subcutaneously injected into the left flank of each mouse and tumours were harvested 30 days after the injection. **B** The volumes of tumours were measured every 5 days. ANOVA. **C** The tumour weight at the 30 days after injection. Mann–Whitney *U* test. **D** Representative images of HE staining and IHC staining with anti-ZRANB1, -SF3B3 or Ki-67 with subcutaneous tumours. **E** A representative image of in vivo footpad model. **F** 5×10^6^ T24 cells were injected into footpad of each mouse and the popliteal LNs were collected 30 days after the injection. **G** The volumes of popliteal LN in the LN metastasis model. **H** HE staining of LN demonstrated the invasiveness of T24 cells with the indicated treatment. Data are shown as mean ± SD (**B**) or min to max (**C**, **G**). *P < 0.05, **P < 0.01^.^ Scale bar = 100 μm.

To validate the ZRNAB1-SF3B3 axis in vivo, we constructed T24 cells in which ZRANB1 was stably knocked down via shRNAs. Cells with or without ZRANB1 knockdown were subcutaneously injected into the left flanks of BALB/c nude mice, and tumour volume was measured every 5 days. The volume and weight of the tumours in the ZRANB1-silenced group were significantly lower than those in the control group (Fig. 7A–C). We also performed IHC staining on tumour tissues from both groups. Not surprisingly, ZRNAB1 expression, SF3B3 expression, and the rate of Ki-67 positivity were lower in the ZRANB1-silenced group than in the NC group (Fig. 7D).

For the LN metastasis model, equal amounts of NC or ZRANB1-silenced T24 cells were injected into the footpads of nude mice, and the popliteal LNs were harvested 30 days later (Fig. 7E). The size and volume of the popliteal LNs in the ZRANB1-silenced group were smaller than those in the NC group (Fig. 7F–G). Moreover, when the LNs were paraffin-embedded and subsequently subjected to HE staining, the results revealed that the degree of LN invasion was greater in the NC group than in the ZRANB1 knockdown group (Fig. 7H). In conclusion, ZRANB1 inhibited the proliferation and LN metastasis of UBC cells in vivo.

## Discussion

Previous studies have identified the deubiquitinase ZRANB1 as a novel oncogenic driver in various malignancies, whose overexpression is correlated with poor survival. For instance, ZRANB1 is highly expressed in hepatocellular carcinoma (HCC) tissues and drives HCC progression by deubiquitinating and stabilising SP1 [28]. It was also reported that ZRANB1 promoted autophagy and suppressed antitumour immunity by protecting cGAS from autophagic degradation [32]. ZRANB1 is generally regarded to specifically hydrolyse both Lys29- and Lys33-linked diubiquitin, but intriguingly, Shan Huang et al. reported that ZRANB1 maintained E3 ligase activity, which was related to its C-terminal operation unit (OUT) domain [21, 27]. In the present study, we clarified the oncogenic role of ZRANB1 in UBC. Our analysis of multiple cohorts (GSE and TCGA) together with clinical validation, revealed that ZRANB1 is significantly upregulated in UBC tissues compared with adjacent normal tissues and that high ZRANB1 expression is correlated with advanced pathological stage, lymph node metastasis, and poor overall survival, which highlights its clinical value as a potential therapeutic target in UBC.

A major novelty of our work lies in the identification of the ZRANB1-SF3B3 axis, which links protein stability to AS regulation. AS is defined as the different combinations of intron-removal and exon-connection during the production of mature RNA from pre-RNA, which is a pivotal posttranscriptional process to expand proteomic diversity [33]. More than 95% of human genes harbour AS events, and it is estimated that each protein-coding gene contains 11 exons and produces an average of 5.4 mRNA transcripts [34–36]. RNA splicing relies on the spliceosome, a large macromolecular complex comprising both RNA and protein, to recognise the junction of introns and exons. Certain *trans*-acting factors and *cis*-acting elements are also involved [37]. The main AS patterns can be divided into five types, of which SE events represent the most prevalent pattern in higher eukaryotes [38]. Abnormal AS activity is generally associated with the occurrence and development of tumours, including various solid tumours as well as haematological malignancies [39–43]. Tumour cells can hijack AS to produce isoforms that favour tumorigenesis, metastasis, anti-apoptosis, chemoresistance, and radioresistance [29, 44–46]. These transcripts can also serve as diagnostic biomarkers and therapeutic targets. One well-known example is that prostate cancer cells utilise AS to escape broadly applied androgen deprivation therapy (ADT). It is widely accepted that prostate cancer is androgen dependent and has antiandrogens, such as enzalutamide, which antagonises the interaction of androgens with androgen receptors (AR), and abiraterone, an inhibitor of androgen biosynthesis, represents the mainstay of treatment for locally advanced or metastatic disease [47]. AR-V7 is a truncated isoform of AR, and it is associated with resistance to ADT and an increased risk of biochemical recurrence after prostatectomy [48]. Compared with wild-type AR, AR-V7 lacks the ligand-binding domain, and this confirmatory change allows persistent AR activation and survival signalling in tumour cells despite the absence of a ligand [49]. Regulation of abnormal AS can be achieved through either interfering with spliceosome components/regulators to modulate splicing efficiency, or by directly abolishing specific isoforms [50]. SF3B1, a component of U2 small nuclear ribonucleoprotein (snRNP), has become a research hit. The prototypic compounds included spliceostatin A, meayamycin B, sudemycins, E7107 and H3B-8800, which mainly affect the assembly of the spliceosome and further impact the AS patterns of a subset of genes [33]. However, few compounds targeting specific transcripts have been utilised in the clinic to date. Risdiplam promotes exon 7 inclusion in SMN2 pre-mRNA and has been approved by the FDA for the treatment of spinal muscular atrophy [51]. Splice-switching antisense oligonucleotides (ASOs) are chemically synthesized short RNA oligos that bind with target pre-mRNAs in a reverse complimentary way to alter the AS mode [50]. We found that ZRANB1 physically interacts with and deubiquitinates SF3B3, thereby protecting it from proteasomal degradation. These findings expand the understanding of how the spliceosome is regulated upstream, suggesting that targeting DUBs such as ZRANB1 could be a strategic approach to destabilise the splicing machinery in cancer cells without directly targeting transcript isoform or the spliceosome itself.

SF3B3, a member of the SF3B complex within the U2 small nuclear ribonucleoprotein (snRNP) complex, plays a crucial role in recognising the branch point sequence of pre-mRNAs and protecting genome stability by facilitating DNA repair [52, 53]. Consistent with our results, overexpression of SF3B3 has been associated with tumorigenesis in colorectal cancer, gastric cancer, hepatocellular carcinoma, oestrogen receptor-positive breast cancer and renal cancer [29, 30, 54, 55]. SF3B3 is involved in the AS of EZH2 pre-mRNA in clear cell renal carcinoma and hepatocellular carcinoma, whereas in colorectal cancer, SF3B3 regulates mTOR exon 8 skipping, leading to lipogenesis via FASN signalling [29, 55]. In our study, SF3B3 knockdown phenocopied the effects of ZRANB1 silencing, suppressing UBC cell viability and invasiveness. Importantly, overexpression of SF3B3 reversed the antitumour effects induced by ZRANB1 depletion, confirming that SF3B3 is a primary downstream effector of ZRANB1. By using next-generation sequencing, we further elucidated that SF3B3 predominantly modulates exon skipping events in UBC, identifying the cell cycle checkpoint kinase CHEK2 as a critical splicing target.

Our mechanistic investigation revealed that SF3B3 is essential for maintaining the expression of full-length CHEK2 while suppressing the exon 4 skipped isoform. It is noteworthy that based on our sequencing results, SF3B3 silencing appears to reduce overall AS events in UBC cells; however, we found that SF3B3 acts as an inhibitor specifically for the AS of CHEK2. One possible explanation would be the dynamic and context-specific control of AS by RNA-binding proteins and other regulatory molecules. The nature of splicing is a competition where the splicing machinery has to discriminate and select between multiple splice sites, involving *cis*-acting sequence features and *trans*-acting factors [56, 57]. CHEK2 is a well-established tumour suppressor that is phosphorylated and activated by ataxia telangiectasia mutated (ATM) during homologous recombination. Its downstream effectors include CDC25C, p53, BRCA1/2 and cyclin D, which further influence DNA repair, cell cycle arrest, apoptosis, senescence, autophagy and ageing. The translation product of the most dominant splicing variant consists of three conserved domains, including a serine–glutamine or threonine–glutamine cluster (SCD) domain at the N-terminus, a forkhead-associated (FHA) domain, and a kinase domain (KD) at the C-terminus. The most highly expressed transcription variant 1 (NM_007194/ENST00000404276.6) consists of 15 exons, and exon 4 lies within the FHA domain. Germline mutations in the CHEK2 gene, represented by c.1100delC and p.I157T, and their association with various cancers have been extensively studied. A germline mutation of CHEK2 was more commonly seen in UBC patients than in controls, yet no significant impact of CHEK2 mutations on overall survival has been observed. Loss of IHC expression of CHEK2 in pT1 UBC was reported to be associated with muscle-invasive progression and worse progression-free survival [58]. However, relevant studies on the AS of CHEK2 in UBC are lacking. Interestingly, our experiments demonstrated that the CHEK2-e4- isoform strongly suppresses UBC proliferation and motility, whereas the full-length protein appears permissive for tumour growth in this context. These findings suggest ZRANB1-SF3B3 axis promotes tumorigenesis by “correcting” splicing to prevent the generation of the antitumour CHEK2-e4- isoform. These findings parallel reports in which cancer cells manipulate splicing factors to shift the balance from pro-apoptotic to anti-apoptotic isoforms; however, the specific molecular mechanisms and the potential clinical relevance require further investigation.

**Fig. 8:**
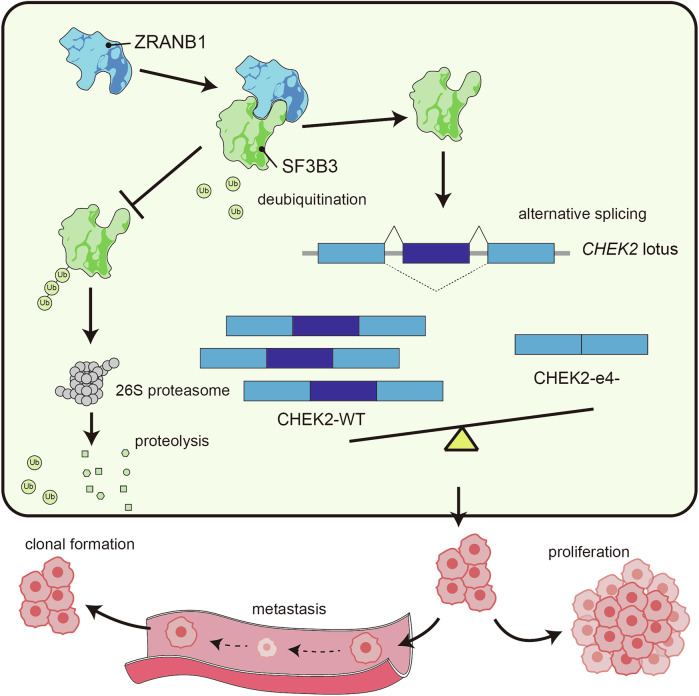
Schematic model of the mechanism underlying the role of ZRANB1 in UBC. ZRANB1 binds with and deubiquitinates SF3B3. SF3B3 plays a role in the AS of CHEK2 transcription.

In conclusion, our study reveals a novel regulatory signalling axis in UBC: ZRANB1 stabilises SF3B3 via deubiquitination, which further inhibits the exon 4 skipping of CHEK2, thereby preventing the expression of a tumour-suppressive isoform. This pathway drives unrestrained proliferation and metastasis in UBC (Fig. 8). These results not only provide new insights into the crosstalk between the UPS and RNA processing but also highlight ZRANB1 as a promising prognostic biomarker and a potential therapeutic target for UBC treatment. Future studies should explore the development of specific small-molecule inhibitors targeting ZRANB1 to disrupt this oncogenic axis.

## Supplementary information

Supplementary figure and table legend

Supplementary Figure 1

Supplementary Figure 2

Supplementary Table 1

Supplementary Table 2

Full and uncropped western blots-1

Full and uncropped western blots-2

Full and uncropped western blots-3

STR profiling report of T24

STR profiling report of UM-UC-3

## Data Availability

The datasets generated during the current study are available in the Gene Expression Omnibus (GEO) [https://www.ncbi.nlm.nih.gov/geo/] and Gene Expression Profiling Interactive Analysis (Gepia) [http://gepia.cancer-pku.cn/].
