# Supplementary material for: ZRANB1 promotes cell proliferation and lymph node metastasis through SF3B3-mediated alternative splicing of CHEK2 in urothelial bladder cancer
Source: Cell Death Dis. 2026 Jun 9;17(1):726. doi: 10.1038/s41419-026-08964-y (PMC13473449; doi:10.1038/s41419-026-08964-y)

Cell Line Authentication Service

STR Profile Report

Sample Submitted By: HUANG LI FANG

Email Address: huanglf2727@163.com

Igeibio Sales Order: IGXN23201

Cell Line Designation: GMC3

Date Sample Received: 2023-08-01

Report Date: 2023-08-04

Methodology:

Twenty-one short tandem repeat (STR) loci plus the gender determining locus, Amelogenin, were amplified using a commercially available STR profiling Kit. The cell line sample was processed using the ABI Prism® 3730xl Genetic Analyzer. Data were analyzed using GeneMapper® ID-X 1.5 software (Applied Biosystems). Appropriate positive and negative controls were run and confirmed for each sample submitted.

Data Interpretation:

Cell lines were authenticated using Short Tandem Repeat (STR) analysis as described in 2012 in ANSI Standard (ANSI/ATCC ASN-0002-2011 *Authentication of Human Cell Lines: Standardization of STR Profiling*) by the ATCC Standards Development Organization (SDO).

Test Results of Submitted Sample:

Alleles / STR 位点	Allele 1	Allele 2	Allele 3	Allele 4
Amelogenin	X	X		
D3S1358	17	18		
vWA	17	17		
D7S820	8	9		
CSF1PO	10	11		
PentaE	8	12		
D8S1179	13	13		
D21S11	31	31		
D16S539	8	9		
D2S1338	23	23		
PentaD	13	13		
D19S433	14	15		
TH01	6	9		
D13S317	8	8		
TPOX	10	10		
D18S51	14	14		
D1S1656	15	17.3		
D5S818	12	12		
D12S391	22	22		
FGA	20	21		

CELL LINE AUTHENTICATION SERVICE

STR Profile Report

Result of searching against igeibio STR Profile Database:

Matches:

NO.	Percent Match	Cell No.	Cell name	D5S818	D13S317	D7S820	D16S539	vWA	TH01	AM	TPOX	CSF1PO
			Query(Your Cell)	12,12	8,8	8,9	8,9	17,17	6,9	X,X	10,10	10,11
1	100%	CRL-1749	UM-UC-3	12,12	8,8	8,9	8,9	17,17	6,9	X,X	10,10	10,11
2	96%	586096	UM-UC-3	12,12	8,8	8,9	8,9	17,17	6,9	X,X	10,10	10,10

NOTE:

Ⅰ. The allele match algorithm compares the 8 core loci plus amelogenin ("AM" for short) only.

Ⅱ. The IGEbio STR Profile Database, which contain more than 8,000 human cell lines' STR profile from Cell Banks, i.e., ATCC, DSMZ, JCRB and RIKEN, and some other publications, is established by IGEbio Biotech Ltd.

Explanation of Test Results:

Cell lines with ≥80% match are considered to be related, i.e., derived from a common ancestry. Cell lines with between a 55% to 80% match require further profiling for authentication of relatedness. Here only show cell lines with ≥80% match.

Disclaimer:

IGE Biotechnology performs STR Profiling following ISO 9001:2008 and ISO/IEC 17025:2005 quality standards. There are no warranties with respect to the services or results supplied, express or implied, including, without limitation, any implied warranty of merchantability or fitness for a particular purpose. IGE Biotechnology is not liable for any damages or injuries resulting from receipt and/or improper, inappropriate, negligent or other wrongful use of the test results supplied, and/or from misidentification, misrepresentation, or lack of accuracy of those results. Your exclusive remedy against IGE Biotechnology and those supplying materials used in the services for any losses or damage of any kind whatsoever, whether in contract, tort, or otherwise, shall be, at IGE Biotechnology's option, refund of the fee paid for such service or repeat of the service.

Contact:

IGE Biotechnology Inc. LTD.
020-89053723
ampseq@igebio.com
Guangzhou, China 510320
http://www.igebio.com

CELL LINE AUTHENTICATION SERVICE

STR Profile Report

Addendum: Electropherogram (peak data) for Submitted Sample

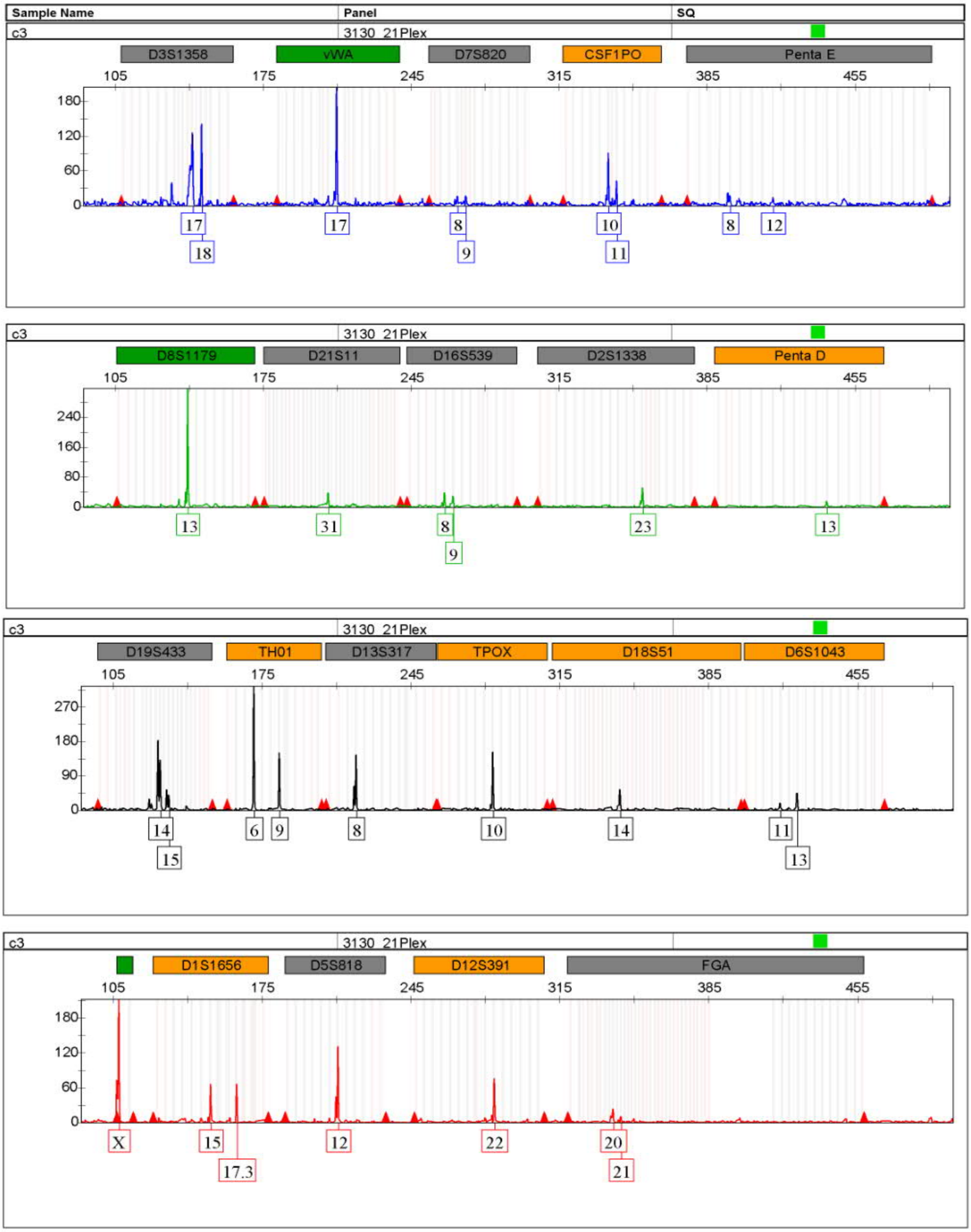

Supplement: Supplementary file 1 [file 41419_2026_8964_MOESM10_ESM.pdf]
