# Supplementary material for: ZRANB1 promotes cell proliferation and lymph node metastasis through SF3B3-mediated alternative splicing of CHEK2 in urothelial bladder cancer
Source: Cell Death Dis. 2026 Jun 9;17(1):726. doi: 10.1038/s41419-026-08964-y (PMC13473449; doi:10.1038/s41419-026-08964-y)
Supplement: Supplementary file 3 [file 41419_2026_8964_MOESM1_ESM.docx]

**Supplementary figure and table legend**

**Supplementary Figure 1 ZRANB1 regulates SF3B3 posttranslationally. A‒B** RT-PCR measured the mRNA level of SF3B3 in T24 and UM-UC-3 cells, when the expression of ZRANB1 was elevated(**A**) or silenced(**B**). **C** NC or ZRANB1-overexpressed UBC cells were subjected to various inhibitors and the expression of SF3B3 was detected by Western blot. Data are shown as mean ± SD. Unpaired, two-tailed student’s *t*-test.

**Supplementary Figure 2 The effect of SF3B3 on different transcripts.** Nucleic acid electrophoresis with specific primers targeting upstream and downstream of exon6 in *LIAS*, exon28 in *DNAH14*, exon2 in *PHF5A*, and exon3 in *CCDC163*, performed with T24 and UM-UC-3 cells.

**Supplementary Table 1 The primers used in this study.**

**Supplementary Table 2 Protein list IP followed by mass spectrometry using anti-flag antibodies and IgG in T24 cells.**
