# Supplementary material for: ZRANB1 promotes cell proliferation and lymph node metastasis through SF3B3-mediated alternative splicing of CHEK2 in urothelial bladder cancer
Source: Cell Death Dis. 2026 Jun 9;17(1):726. doi: 10.1038/s41419-026-08964-y (PMC13473449; doi:10.1038/s41419-026-08964-y)
Supplement: Supplementary file 4 [file 41419_2026_8964_MOESM4_ESM.docx]

**Supplementary Table 1**

The primers used in this study.

|  | **Sequence (5’-3’)** |
| --- | --- |
| **Primers** |  |
| CHEK2 forward | TTGCTTTGATGAACCACTGCTG |
| CHEK2 reverse | GAAAGCCAGCTTTACCTCTCCA |
| DNAH14 forward | CCAGGACAAGTGGTACTTACTGT |
| DNAH14 reverse | AAGCTGGCATTTCCTTGAGACA |
| LIAS forward | TCCTGACATCTGTGGATCGAGA |
| LIAS reverse | CTATTGCTTTGAGATCACCTCGA |
| CCDC163 forward | CTGTTTCTTCTGTAGCAGTGGC |
| CCDC163 reverse | GCATGGAGCTGAAAGATGCTG |
| PHF5A forward | CCTGATTTGATCTTTTGCCGCA |
| PHF5A reverse | CTGGATGGTGCACTCCTTACAA |
